# Assessment of Feeding Behaviors and Parents’ Frustrations of Children with Autism Spectrum Disorder in Lebanon: A Case-Control Study

**DOI:** 10.3390/children10010117

**Published:** 2023-01-05

**Authors:** Melissa Rouphael, Batoul Hojeij, Diana Ezzedine, Hussein Mortada, Yonna Sacre, Tania Bitar, Elissa Naim, Walid Hleihel, Maha Hoteit

**Affiliations:** 1 Department of Biology, Faculty of Arts and Sciences, Holy Spirit University of Kaslik, Jounieh P.O. Box 446, Lebanon; 2 Faculty of Public Health, Section 1, Lebanese University, Beirut P.O. Box 6573, Lebanon; 3 Faculty of Science, Lebanese University, Zahle 6573, Lebanon; 4 Department of Nutrition and Food Sciences, Faculty of Arts and Sciences, Holy Spirit University of Kaslik (USEK), Jounieh P.O. Box 446, Lebanon; 5 Institut National de Santé Publique, Epidémiologie Clinique et Toxicologie (INSPECT-LB), Beirut 1100, Lebanon; 6 School of Medicine and Medical Sciences, Holy Spirit University of Kaslik, Jounieh P.O. Box 446, Lebanon; 7 PHENOL Research Group (Public HEalth Nutrition prOgram Lebanon), Faculty of Public Health, Lebanese University, Beirut P.O. Box 6573, Lebanon; 8 Lebanese University Nutrition Surveillance Center (LUNSC), Lebanese Food Drugs and Chemical Administrations, Lebanese University, Beirut P.O. Box 6573, Lebanon

**Keywords:** autism spectrum disorder, eating habits, feeding behaviors, parents, typically developing children

## Abstract

Children with autism spectrum disorder (ASD) exhibit restrictive and repetitive behaviors that affect their eating habits. The purpose of this study is to identify the behavioral feeding problems and eating habits among ASD children compared to typically developed (TD) children age/gender-matched controls, along with their parents’/caregivers’ strategies for dealing with them. It included 43 ASD children and 43 TD children aged two to eleven years. The analysis was performed based on two valid questionnaires: the Behavior Pediatrics Feeding Assessment Scale (BPFA) and “My Child Eating Habits” (MCEH). The BPFA and MCEH scores conceded three manifestations that fall into food selectivity and problematic mealtime behavior in both groups of children. Compared to TD children, children with ASD exhibited higher BPFA scores, which indicated food-related behavioral and skill-based problems (*p* = 0.004). Children with ASD were less likely to consume fruits, vegetables, and milk than TD children, which may lead to nutritional deficiencies (*p* = 0.003, *p* = 0.003, and *p* = 0.010, respectively). Parents of ASD children were concerned about their behavioral problems and expressed their intention of an early intervention. These findings highlight the importance of nutritional clinical routines that incorporate the evaluation of the nutritional status and feeding behaviors of ASD children.

## 1. Introduction

According to the Diagnostic and Statistical Manual of Mental Disorders (DSM-5), autism spectrum disorder (ASD) is a lifelong neurodevelopmental disorder characterized by deficits in social interaction and communication skills along with repetitive, restricted, and stereotyped behaviors and activities [1]. The prevalence of ASD has intensely increased over time and is currently estimated to affect 1 in 100 children around the world, with a male-to-female ratio of 4:1 [2]. However, this incidence is substantially greater in Lebanon, where it is estimated to affect 1 in 66 children in the Beirut and Mount Lebanon regions [3]. This rise in its prevalence suggests the need for a deeper understanding of the etiology of this disorder since the exact cause of ASD remains largely unclear. However, several studies have shown that ASD is a multifactorial disorder where genetic, epigenetic, and environmental factors contribute to the risk of developing ASD [4,5,6,7].

In addition to the core symptoms, children with ASD frequently experience sensory sensitivities, such as hypo or hypersensitivity to light, sound, taste, smell, texture, and touch [8]. These abnormalities are often associated with different comorbidities such as disruptive behaviors, gastrointestinal symptoms, and eating problems [9]. Indeed, feeding problems have been identified early in the history of ASD research [10] and continue to be reported in many children with ASD [11,12]. Several studies reported that children with ASD experience about five times more feeding problems than typically developing children [8,13]. Food selectivity, food refusal, and disruptive mealtime behaviors are widely recognized as problematic for autistic children [14]. They often appear in the toddler and preschool years and persist until adolescence [12]. Therefore, children with ASD are at a higher risk of nutritional deficits resulting from limited dietary variety. They are also at a higher risk of developing medical and developmental problems, including malnutrition, suboptimal growth, social deficits, and poor academic progress [15].

Moreover, feeding problems in children with ASD are significantly associated with stress in parents. Parental stress may be improved by teaching parents strategies to improve eating and decrease disruptive mealtime behaviors [16]. Thus, the diagnosis and treatment of these feeding problems are essential for the best interests of the child and the family members [17]. Hence, the investigation of the feeding indicators and patterns through routine care, starting from the diagnosis of ASD and expanding throughout the child’s growth, should be implemented frequently because the limited number of published studies in Lebanon yielded irreconcilable results.

This study aims to identify feeding behavioral problems and eating habits in children with ASD when compared to the matched controls, as well as the impact of feeding problems on their caregivers.

## 2. Materials and Methods

### 2.1. Study Design

This was a case–control study conducted on 86 Lebanese children during a period of 8 months from September 2017 to May 2018. The sample was split into two groups (case/control 1:1), an ASD group and a control group.

### 2.2. Study Sample

A sample of 43 children with ASD and 43 healthy children participated in this study. Children with ASD were recruited from specialized institutions in Beirut, whereas children considered to be typically developing children (TD) were recruited from nursery and elementary schools around Beirut. These children, according to their caregivers and based on their medical history, had not experienced delays in motor and language development or behavioral problems. They matched the age bracket and gender of the enrolled children with ASD. The inclusion criteria were (1) children aged between 2 and 11 years, (2) the diagnosis of ASD was based on the criteria for autism defined in the Diagnostic and Statistical Manual of Mental Disorders, Fifth Edition (DSM-5) as well as on the Childhood Autism Rating Scale (CARS), and (3) informed consent of the child’s caregiver to participate in the study was obtained. The exclusion criteria were (1) children with down syndrome and fragile X syndrome, and (2) children treated for non-communicable diseases, cancer, or any other diseases that could affect their feeding behavior and eating habits.

### 2.3. Measurement Tools

#### 2.3.1. Anthropometric Measurement

The body mass index was calculated and computed via Statistical analysis was performed using the Statistical Package of Social Sciences Software (SPSS) (Version 24.0. IBM Corp: Armonk, NY, USA). The references employed are based on the BMI cut-off points to the age plotted on CDC gender-specific charts (Clinical growth charts, Center for disease control and prevention.2017. https://www.cdc.gov/growthcharts/clinical_charts.htm/ accessed on 21 September 2022. Growth Charts—Clinical Growth Charts, 2019).

$$BMI=\frac{weight\;\left(\mathrm{kg}\right)}{\;height^{2}\;\left(m^{2}\right)}$$

#### 2.3.2. Socio-Demographic Assessment

The caregivers involved in this study were asked to fill out a socio-demographic questionnaire. They were asked to report the child’s age, parent’s education level, parent’s employment status, monthly income, number of children in the family, as well as whether the child follows a specific diet, and follow up.

#### 2.3.3. Feeding and Eating Patterns Assessment Tools

##### Behavior Pediatric Feeding Assessment Scale (BPFA)

Information on the mealtime and feeding behaviors was gathered using the “Behavior Pediatric Feeding Assessment Scale” (BPFA), a parent report tool. This scale consists of 35 items. The first 25 items address the child’s behavior, and the last 10 items address the parent’s feelings about the child’s behavior and the parent’s strategies for coping with their child’s feeding problems [18]. Using a Likert scale from one to five, the parents were asked to indicate how frequently the behavior occurs for each of the 35 questions. After rating the behavior, they were asked to report whether they felt the mealtime behavior is problematic using a dichotomous scale (0 = no and 1 = yes). This results in two different scores, the total frequency score (TFS) (maximum score 175) and the total problem score (TPS) (maximum score 35), respectively. The frequency scores reflect how often a behavior occurs, and the problem score represents the number of problematic feeding behaviors. Individuals with scores above 84 for the TFS and 9 for the TPS are considered to be at risk of feeding problems [19]. 

Crist and Napier Philips proposed the five following factors: picky eaters, toddler refusal—general, toddler refusal—texture foods, older children refusal—general, and stallers. Allen et al., examined the factor structure and validity of the Behavioral Pediatrics Feeding Assessment Scale in preschoolers with autism spectrum disorder. A categorical exploratory factor analysis (CEFA) was conducted and identified a three-factor model: mealtime behavior, medical or oral difficulties, and food acceptance [20].

##### My Child Eating Habits Scale (MCEH)

The purpose of the Eating Habits Questionnaire is to compare feeding difficulties among children without autism and those with autism. It includes 24 items about the nature of feeding issues, food preference, food refusal, potential causes of the child’s eating habits, the specific concerns of parents, and their willingness to seek feeding therapy. Parents were asked to rate each item on a 5 point scale of (1) strongly disagree, (2) disagree, (3) undecided, (4) agree, and (5) strongly agree [21].

### 2.4. Statistical Analysis

Data were analyzed using the Statistical Package for Social Sciences Statistical analysis was performed using the Statistical Package of Social Sciences Software (SPSS) (Version 24.0. IBM Corp: Armonk, NY, USA). Before all analyses, the normality of the population was assessed through the Shapiro–Wilktest. A *p*-value of <0.05 was considered significant. The data are shown as the mean ± standard deviation. The statistical tests carried out were the independent sample Mann–Whitney test to compare the mean and standard deviation of the continuous variables between the two groups, and the Chi-Square was performed to assess the relationship between the two categorical variables.

### 2.5. Ethical Considerations

The study was performed following the ethical standards as laid down in the 1964 Declaration of Helsinki and its later amendments, the Lebanese University Ethical Committee approved the study protocol (CU#13; 29 July 2017), and all the parents of the participants provided informed consent.

## 3. Results

### 3.1. Description of the Study Sample

The final sample included 43 ASD and 43 TD children with a mean age of (5.9 ± 2.6) and (6.2 ± 2.5) years, respectively. The general characteristics of the sample are shown in Table 1.

No significant differences were observed in the mean age, parents’ education status, monthly income, or being the only child in the family between the two groups. However, only ASD children were on a special diet (*p* < 0.01) and on a follow-up (*p* < 0.01) compared to TD children. TD children were more likely to have more family members than ASD children (5 ± 1.1 and 4.5 ± 1, respectively, *p* = 0.025) (Table 1). Our results also illustrated no statistical differences in the body mass index (BMI) between the two groups (*p* > 0.05), Table 1. However, the total prevalence of overweight and obesity was higher in ASD children than TD children (58% and 44%, respectively).

Furthermore, a Pearson Chi-square was calculated for the two groups to explore the impact of the monthly income and the parents’ education status on the BMI. Our results showed no significant differences in the monthly income (*p* = 0.40 and *p* = 0.17, respectively), the father’s education level (*p* = 0.65), or the mother’s education level (*p* = 0.48), respectively, according to the BMI. The findings also revealed that the educational background of the parents was not an incentive for putting ASD children on diets or a follow-up (*p* < 0.05), (Data not shown).

### 3.2. BPFA Scale Assessment

According to the BPFA’s score, ASD children were less likely to eat fruits, vegetables, and drink milk than TD children (*p* = 0.003, *p* = 0.003, *p* = 0.01, respectively) and were more likely to choke at mealtimes (*p* = 0.003). However, ASD children did not statistically differ from TD children in drinking rather than eating and eating only soft foods (*p* = 0.134 and *p* = 0.590, respectively). Moreover, our results showed that ASD children were more likely to get up from the table during mealtimes than TD children (*p* = 0.000) (Table 2), which was considered a major problem for the parents (55.8%), and they were less likely to delay eating by talking than controls (*p* = 0.000).

The parents’ strategies for dealing with food refusal, such as serving something else, coaxing the child to get them to take a bite, and putting the food in the child’s mouth by force if the child refuses to eat, were significantly higher among the parents of ASD children (*p* = 0.026, *p* = 0.021, and *p* = 0.046, respectively). The occurrence of parents getting frustrated and/or anxious when feeding their children and getting angry with their children at mealtimes was significantly higher among the parents of ASD children (*p* = 0.002 and *p* = 0.054, respectively), Table 2.

The mean TFS was higher for ASD compared to the control group (76.35 ± 16.7 and 65.53 ± 12.6, respectively, *p* = 0.004), indicating that higher levels of problematic feeding behavior and the mean TPS was higher for ASD parents than for the control parents (10.7 ± 6.5 and 5.3 ± 5.1, respectively, *p* < 0.000). Hence, almost all of the questioned items are considered to be a problem for parents of ASD children. Given the established cut-offs, which are indicative of feeding problems, the prevalence of feeding problems was 30% in ASD children, which was six times more than the control group (5%; *p* = 0.002), thereby warranting a nutritional intervention (according to TFS) and 58%, which was almost three times higher than the controls (23%, *p* = 0.001) (according to TPS). The relevant results are presented in Table 3.

When comparing the genders within the ASD group, girls were more likely to spit food (*p* = 0.016), whereas boys were more likely to get up from the table during mealtimes (*p* = 0.039). However, no gender difference in the total frequency score was presented (*p* = 0.795). Moreover, within the age categories, ASD children aged 6 years and younger were more likely to take longer than 20 min to finish their meals and to drink milk than those older than 6 years (*p* = 0.037 and *p* = 0.006, respectively), whereas the latter group was more likely to have their parents serve something else when they did not like what they were served (*p* = 0.04) (data not shown).

Our results illustrated that the mealtime behavior was significantly different with a higher value for TD children than ASD (23.9 ± 3.4 and 20.9 ± 4.8, respectively, *p* = 0.003), Table 4.

The five interpretable factors proposed by Crist & Phillips (2001) [18] showed no difference between the ASD and control group except for the picky eater factor, which was higher among TD children than ASD children (34.4 ± 4 and 32.2 ± 4.6, respectively, *p* = 0.028), Table 5.

### 3.3. MCEH Scale Assessment

Our results showed no statistical difference between the two groups in terms of a refusal of or preference for food based on the presentation (*p* = 0.441), color (*p* = 0.814), texture (*p* = 0.4), crunchiness (*p* = 0.234), smoothness (*p* = 0.851), temperature (*p* = 0.632 for cold and *p* = 0.204 for hot), and negative experience with food (*p* = 0.254), Table 6. 

However, ASD children were significantly more likely to reject particular foods (*p* = 0.001) and their parents reported that the majority of children would consistently choose the same foods and drinks during meals (*p* = 0.011). Moreover, the results showed that the parents of ASD children were more concerned about their child’s eating habits than the control group (3.16 ± 1.511 and 2.26 ± 1.136, respectively, *p* = 0.005). Indeed, they were more likely to consider their child’s eating habits a problem (*p* = 0.004) and they also emphasized the importance of an important intervention for the child’s eating habits more than the control group (3.16 ± 1.252 and 2.51 ± 1.222, respectively, *p* = 0.016).

Furthermore, there was no significant difference in the mean responses for any questionnaire item between the genders or between the two age categories (data not shown).

## 4. Discussion

To our knowledge, this study is the first to examine the feeding behavior and eating habits of Lebanese ASD children compared to typically developing children and to identify the impact of feeding problems on their caregivers. Overall, the findings are encouraging and may help prevent and treat nutritional deficiencies in Lebanese autistic children as well as enhance the quality of their diet.

Our results showed that the prevalence of an unhealthy weight is significantly higher among children with ASD compared to the matched controls. Previous studies reported that overweight and obesity found among children are related to dietary factors including a high energy intake, fast food consumption, sugary beverages, and snack foods, and all of these behaviors are present in autistic children as well as in typically developing children [22,23]. Furthermore, a reduced activity and increasing sedentary behavior are two of the main contributors towards weight gain and a high BMI. Children with ASD are known to have motor impairments, which can affect their ability to participate in sports and physical activities. Such motor impairments include decreased muscle tone, oral motor problems, postural instability, and poor motor skills [24]. In addition, children with ASD engage in fewer physical activities due to the impairments in their social skills, which may limit their participation in structured activities with peers [25]. Moreover, psychotropic medications that may be prescribed for the management of autism present a risk factor for weight gain among this group [26]. Furthermore, children with ASD may also have genetic risk factors for obesity, such as 11p14.1 or 16p11.2 microdeletions [27,28]. In addition, it has been shown that high BMI levels are associated with adverse health outcomes, including insulin resistance, heart disease, diabetes, and sleep-disordered breathing [29]. Childhood obesity is also associated with a family economic burden and can negatively impact physical, emotional, and social functioning, as well as academic achievement, which could exacerbate an ASD-related disability and result in a lower quality of life [30]. ASD children who are overweight or obese are more likely to be bullied and socially isolated [29].

Our findings suggest that feeding problems are significantly more prevalent in ASD children than in the control group. This conformed with other studies where the prevalence of feeding problems among ASD children were found to range from 56 to 87% [15,31]. Children with ASD may have gastrointestinal, anatomical, metabolic, motor, or sensory feeding issues [32]. These problems could also result from a limited ability to communicate or from poor social and cognitive skills [12]. According to the research findings, children with ASD consume fewer types of food than normally developing children and exhibit selective feeding five times more frequently [13]. In the present study, ASD children preferred starches and snack items, with the least preferred food group being fruits and vegetables. These findings are consistent with other studies that assessed the dietary intake of ASD children, reporting that they consume fewer than the recommended servings of fruits and vegetables, yet have a carbohydrate intake within the normal range and shifting towards the upper ends [33,34,35]. This food selectivity can have serious consequences, such as malnutrition and micronutrient deficiencies. Malnutrition predisposition may result from the parents’ beliefs regarding nutrition, abnormal feeding practices, restricted and repeated eating habits, restrictive diet therapies, and medication use that children are frequently exposed to and affected by [36]. Moreover, this eating pattern might be related to sensory selectivity in children with autism [37]. Therefore, restricting their food intake may lead to nutritional inadequacies and insufficiencies whilst also having a high BMI [38], especially if this restriction for specific food types and varieties is consistent. Indeed, a nutrient deficiency in ASD children was observed in specific vitamins and minerals, with a positive correlation between the serum levels of magnesium, iron, calcium, vitamin B12, and folate and their levels in the food intake [39], in addition to a significantly lower protein intake in ASD children compared to typically developing children [40]. As a result, food selectivity may pose a health risk. High carbohydrate intakes in children can be detrimental since they lack the nutritional value of these foods, which replace other nutritious foods. Carbohydrates can also cause constipation, while also affecting one’s appetite and food intake. Moreover, an increased intake of these carbohydrates combined with a decreased intake of protein and fiber can lead to fluctuations in the blood sugar levels, thus affecting the child’s mood and concentration [41].

Children with ASD have been found to have certain feeding difficulties, mainly a food refusal and selectivity according to their organoleptic properties [14]. This sensory food aversion is presented by preferring food based on its temperature, color, taste, smell, and/or texture and ranges from mild to severe [37]. Our findings are in line with other research that found no differences in food refusal or the preference between children with ASD and TD based on the color, texture (smooth or crunchy), temperature (hot or cold), and presentation of the food [38,42]. On the contrary, Huxham et al. [41] have found that the majority of participants readily ate crunchy, dry foods as well as smooth, puréed foods, indicating that a food’s texture played a significant impact on participants’ meal preferences.

Furthermore, ASD children exhibited more mealtime behavior problems than TD children [43]. Indeed, they were more aggressive during mealtime, such as gagging and spitting out food, which was in agreement with what was found in Margari et al.’s study. Some gender differences were observed among autistic children in certain mealtime feeding behaviors (spitting food and getting up from the table during mealtime). Hence, the mealtime behavioral difficulties presented in ASD children can be related to food phobia and the child’s pragmatic deficits, where the deficits in social interaction may affect the child’s learning of what constitutes appropriate mealtime behavior [43,44]. In addition, problems with chewing food were greater among the ASD group, which is related to the motor function among these children [45].

These mealtime behavioral problems are concomitant with the feeding difficulties presented by the child and parent strategies against these behaviors [44]. However, parental strategies for dealing with these issues create a tipping point in children’s behavior. Families ultimately prepare meals based on their child’s acceptance because children frequently exhibit food refusal and sensitivity behaviors and are particularly confined to certain foods [46]. In our study, the parents of ASD children were presenting food that their children would accept or would make something else if he/she did not like what was served. Hence, these family practices are positively reinforcing a food refusal in ASD children [47]. Consequently, these feeding behavior and eating habits problems result in family stress, fatigue, and frustration and they also influence the family’s diet [48]. Moreover, our study has found that the parents of children with ASD had a higher prevalence of frustration, anger, and anxiety when feeding their children. Parental mental health problems negatively affect the adherence to treatment recommendations for children with autism [46]. Furthermore, they reported that they would force their children to eat when necessary. This might be explained by the motivational and behavioral difficulties presented in children with ASD that in turn lead to unintended parental enforcement, which may lead to food refusal and selectivity [46]. This highlights the importance of providing counseling and training for the parents of ASD children [46]. The Autism MEAL Plan [49] and the Behavioral Parent Training Program for Feeding Problems [17] are examples of the models developed as treatment strategies for selective eating among ASD children up to eight years of age. Moreover, recently, Kuschner et al. [50] developed the BUFFET program that leverages cognitive behavioral treatment strategies to deal with selective eating among youths with ASD by helping them to "develop skills to cope with anxiety, and to think and act flexibly with new or non-preferred foods". Two advantages of this program are the high attendance rate and parent satisfaction, which make it feasible to be a promising treatment for outpatient selective eating in older youths with ASD.

Although various studies have demonstrated the existence of feeding and feeding behavior problems in children with autism, few studies have focused on the parental strategies and willingness to seek interventions to address these problems. Our study found that parents of children with autism were highly concerned about their children’s eating habits, viewed them as problematic, and reported that these eating habits required treatment. This highlights the importance and potential of initiating nutrition-based interventions and outpatient models to address the issues presented alongside other medical and behavioral therapies.

This study has limitations that warrant mention. First, the small sample size has limited the ability to detect significant differences between the two groups and has affected the generalizability of our findings. Second, the sample was not fully representative, as the vast majority of participants were recruited from the Beirut and Mount Lebanon region. Thus, the results cannot be generalized to the whole country. Third, the instrument used was a self-administered questionnaire based on the caregiver’s responses. This may raise questions regarding the quality since the participants may answer quickly, dishonestly, or inattentively. Thus, not having direct information from the children might cause a small bias in the results. Fourth, the administered drugs were not evaluated in this study. It would have been of great value if the dietary intake of these children had been assessed to have a more accurate view of these behaviors and habits. Further research is also needed to determine the long-term health burden of severe food selectivity as well as the impact on the caregiver, taking into account a larger sample covering all the districts in Lebanon. In addition, due to the unavailability of the tools in the Arabic language, we translated the questionnaires used in this study to Arabic that were reviewed by experts. Despite all of these limitations, this study was the first to address the feeding behaviors and eating habits of autistic children in Lebanon, which may be the basis for future research and interventions in the country.

## 5. Conclusions

This study has identified common feeding difficulties and eating habits in children with ASD and assessed their impact on the caregivers. Most ASD and TD children presented a normal range of BMI for their age, but a higher number of ASD children were overweight. There was no difference in the food preference or refusal based on the sensory characteristics (presentation, color, texture, and temperature) among both groups. However, children with ASD were less likely to consume fruits, vegetables, and milk than TD children, which may lead to nutritional deficiencies. Most mealtime behavior problems were higher among children with autism, and some gender differences in committing these behaviors were presented. Moreover, the parents of ASD children were concerned about these problems, thus expressing their intention of an early intervention in dealing with their children’s eating habits. Therefore, these findings highlight the importance of nutritional clinical routines that incorporate the evaluation of the nutritional status and feeding behaviors in ASD children.

## Figures and Tables

**Table 1:** Socio-demographic and anthropometric characteristics of ASD and TD children.

Variable	TD Children	ASD Children	*p*-Value ^a^
Gender (*n* = 43)			-
Male	35	35	
Female	8	8	
Age ^a^ (mean years ± SD)	6.2 ± 2.5	5.9 ± 2.6	0.472
Mother education level			
<secondary degree	11 (26%)	7 (16%)	0.289
≥secondary degree	32 (74%)	36 (84%)	
Father education level			
< secondary degree	18 (42%)	21 (49%)	0.516
≥ secondary degree	25 (58%)	22 (51%)	
Monthly income			
<1,500,000	18 (42%)	24 (56%)	0.196
≥1,500,000	25 (58%)	19 (44%)	
Child on special diet	0 (0%)	10 (23%)	0.001
Child on follow **up** ^b^	0 (0%)	21 (49%)	0.000
Child as the only child in family	4 (9%)	7 (16%)	0.333
Family members (mean ± SD)	5 ± 1.1	4.54 ± 1	0.025
Anthropometric characteristics			
BMI for age	**Mean (SD)**	**Mean (SD)**	***p*** **-Value ^a^**
			0.306
Underweight	3 (7%)	3 (7%)	
Normal	21 (49%)	15 (35%)	
Overweight	6 (14%)	13 (30%)	
Obese	13 (30%)	12 (28%)	

*Note.* ASD: autism spectrum disorder, TD: typically developing, SD: standard deviation. ^a^ Age breakdown among children with ASD: 27 (63%) 3–6 years, 16 (37%) 6–12 years among TD children; 26 (61%) 3–6 years, 17 (39%) 6–12 years among ASD children. ^b^ *p*-value for differences between ASD and TD children from Chi-square test for categorical variables and independent *samples Mann–Whitney U test* for continuous variables. BMI: body mass index. ASD: autism spectrum disorder. TD: typically developing. ^a^ *p*-value for differences between ASD and TD children from Chi-square test. ^b^ behavioral and nutritional follow up.

**Table 2:** Mean BPFA scores related to parents’ attitudes and food-related behaviors for ASD and TD children.

Questionnaire Item	TD Controls ^a^ Mean ± SD	ASD ^b^ Mean ± SD	*p*-Value ^c^
Child eats fruits.	4.16 ± 1.07	3.19 ± 1.55	0.003
Child has problems chewing food.	1.44 ± 1.01	2.19 ± 1.45	0.007
Child enjoys eating.	3.86 ± 1.10	3.91 ± 1.09	0.825
Child chokes or gags at mealtime.	1.07 ± 0.34	1.63 ± 1.16	0.003
Child will try new foods.	3.2 ± 1.21	2.84 ± 1.29	0.149
Child eats meat and/or fish.	4.21 ± 1.19	3.79 ± 1.36	0.112
Child takes longer than 20 min to finish a meal.	2.51 ± 1.20	2.72 ± 1.24	0.380
Child drinks milk.	3.67 ± 1.21	2.74 ± 1.73	0.010
Child comes readily to mealtime.	4.16 ± 1.07	3.98 ± 1.19	0.480
Child eats junky snack food but will not eat at mealtime.	2.02 ± 1.08	2.33 ± 1.21	0.239
Child vomits just before, at, or just after mealtime.	1.16 ± 0.69	1.40 ± 1.00	0.177
Child eats only ground, strained or soft food.	1.86 ± 1.39	1.91 ± 1.31	0.590
Child gets up from table during mealtime.	2.12 ± 1.18	3.28 ± 1.52	0.000
Child lets food sit in his/her mouth and does not swallow it.	1.23 ± 0.57	1.47 ± 0.86	0.228
Child whines or cries at feeding time.	1.47 ± 0.91	1.60 ± 0.98	0.393
Child eats vegetables.	4.09 ± 1.11	3.07 ± 1.64	0.003
Child tantrums at mealtimes.	2.09 ± 1.13	2.12 ± 1.14	0.956
Child eats starches (for example potato, noodles).	4.37 ± 0.93	4.12 ± 1.22	0.375
Child has a poor appetite.	2.23 ± 1.21	2.42 ± 1.35	0.620
Child spits out food.	1.49 ± 0.94	2.05 ± 1.11	0.008
Child delays eating by talking.	2.21 ± 1.19	1.42 ± 0.91	0.000
Child would rather drink than eat.	2.05 ± 1.13	2.53 ± 1.45	0.134
Child refuses to eat meals but requests food immediately after the meal.	1.95 ± 1.15	1.58 ± 1.07	0.076
Child tries to negotiate what s/he will eat and what s/he will not eat.	2.47 ± 1.32	2.12 ± 1.40	0.186
Child has required supplemental tube feeds to maintain proper nutritional status.	1.09 ± 0.43	1.05 ± 0.31	0.559
Parent gets frustrated and/or anxious when feeding his/her child.	1.56 ± 0.91	2.47 ± 1.45	0.002
Parent coax child to get him/her to take a bite.	2.26 ± 1.22	3.02 ± 1.58	0.021
Parent uses threats to force child to eat.	1.42 ± 0.70	1.30 ± 0.80	0.167
Parent feels confident that his/her child gets enough to eat.	3.88 ± 1.31	3.91 ± 1.32	0.856
Parent feels confident in his/her ability to manage child’s behavior at mealtime.	3.84 ± 1.13	3.65 ± 1.21	0.484
If the child does not like what is being served, parent makes something else.	2.74 ± 1.38	3.44 ± 1.37	0.026
When the child has refused to eat, the parent has put the food in child mouth by force if necessary.	1.16 ± 0.49	1.53 ± 0.98	0.046
Parent disagree with other adults about how to feed child.	2.37 ± 1.42	1.95 ± 1.00	0.236
Parent feels that his/her child patterns hurt his/her general health.	1.79 ± 1.17	2.40 ± 1.38	0.037
Parent gets so angry with child at mealtimes that it takes him/her a while to calm down after the meal.	1.23 ± 0.57	1.63 ± 1.02	0.054

*Note*. BPFA: Behavior Pediatric Feeding Assessment. ASD: autism spectrum disorder. TD: typically developing. ^a, b^ Total number of responses for each item is 43. ^c^ *p*-value for differences between ASD and TD controls from independent sample *t*-test.

**Table 3:** Frequency and problem score of BPFAS.

Scores	TD Controls Mean ± SD	ASD Mean ± SD	*p*-Value *
Total frequency scores	65.53 ± 12.625	76.35 ± 16.656	0.004
Total problem scores	5.26 ± 5.081	10.70 ± 6.479	<0.000
TFS score by cut-off Higher than normative value (>84)	2 (5%)	13 (30%)	0.002
TPS score by cut-off Higher than normative value (>9)	10 (23%)	25 (58%)	0.001

Values are mean ± SD for numerical variables. N (%) for categorical variables. * *p*-value for differences between ASD and TD controls from independent sample *t*-test and Chi-square.

**Table 4:** The mean scores of the three factors model proposed by Allen et al., (2015) between the ASD and control group.

		TD Controls (Mean ± SD)	ASD (Mean ± SD)	*p*-Value ^a^
**Allen three factor model**	Mealtime behavior	23.9 ± 3.4	20.9 ± 4.8	0.003
	Food acceptance	26.6 ± 5.3	27.3 ± 5	0.585
	Medical/oral motor	20.2 ± 3.4	21.3 ± 4.2	0.252

*Note.* ASD: autism spectrum disorder. TD: typically developing. SD: standard deviation. ^a^ *p*-values for differences between ASD and TD children from independent samples Mann–Whitney *U* test to compare the means between groups.

**Table 5:** Mean scores of the five interpretable factors proposed by Crist and Phillips (2001) between the ASD and control group.

		TD Controls (Mean ± SD)	ASD (Mean ± SD)	*p*-Value ^a^
**Crist five interpretable factors**	Picky eaters	34.4 ± 4	32.2 ± 4.6	0.028
	toddler refusal—general	27.1 ± 5.4	28.3 ± 5.4	0.350
	Toddler refusal—texture foods	21.3 ± 3.2	22.3 ± 4.1	0.255
	Older Children refusal—general	37.0 ± 5.0	37.4 ± 5.1	0.849
	Stallers	34.1 ± 4.9	35.6 ± 5.3	0.277

*Note.* ASD: autism spectrum disorder. TD: typically developing. SD: standard deviation. ^a^ *p*-values for differences between ASD and TD children from independent samples Mann–Whitney *U test* to compare the means between groups.

**Table 6:** Mean MCEH scores related to parent’s responses among ASD and TD groups.

Questionnaire Item	TD Mean ± SD	ASD Mean ± SD	*p*-Value
(1) I consider my child’s eating habits a problem.	2.40 ± 1.072	3.12 ± 1.219	0.004
(2) My child has few foods they willingly accept.	3.02 ± 1.263	3.53 ± 1.141	0.062
(3) My child prefers foods that are crunchy (cereal, chips, crackers).	3.16 ± 1.174	3.49 ± 1.032	0.234
(4) My child prefers foods that are smooth (yogurt, applesauce, pudding).	2.81 ± 1.052	2.86 ± 1.187	0.851
(5) My child prefers foods that are hot.	3.02 ± 1.012	2.72 ± 1.008	0.204
(6) My child prefers foods that are cold.	3.05 ± 0.925	3.19 ± 0.932	0.632
(7) My child has difficulties accepting new foods.	2.74 ± 1.093	3.19 ± 1.2	0.092
(8) My child refuses particular foods and/or food groups (meats, vegetables, etc.).	2.28 ± 0.984	3.14 ± 1.146	0.001
(9) My child refuses foods/drinks based on the presentation (particular bowl, utensil).	2.47 ± 1.120	2.65 ± 1.152	0.441
(10) My child refuses foods/drinks based on the color.	2.49 ± 1.222	2.40 ± 1.137	0.814
(11) My child refuses foods based on the texture (smooth/crunchy, soft/hard).	2.53 ± 1.141	2.74 ± 1.071	0.400
(12) My child refuses foods/drinks based on a prior negative experience with that item (choking, stomach ache, vomiting).	2.33 ± 1.248	2.56 ± 1.098	0.254
(13) My family’s diet has changed as a result of my child’s eating habits.	2.26 ± 1.026	2.49 ± 0.985	0.321
(14) I only present foods/drinks I know my child will accept.	2.33 ± 1.190	2.98 ± 1.185	0.011
(15) I present new foods/drinks to my child even if they have previously refused that item.	3.26 ± 1.093	3.79 ± 0.861	0.028
(16) I am concerned about my child’s eating habits.	2.26 ± 1.136	3.16 ± 1.511	0.005
(17) I am concerned with my child’s nutrition based on their eating habits.	2.26 ± 0.978	3.00 ± 1.345	0.006
(18) I am concerned with my child’s ability to socialize based on their eating habits.	2.02 ± 0.988	3.05 ± 1.174	0.000
(19) I am concerned with stress my child’s eating habits have caused my family.	2.60 ± 4.821	2.74 ± 1.177	0.001
(20) I am more concerned now with my child’s eating habits than I have been in the past.	2.12 ± 1.138	2.67 ± 1.063	0.005
(21) I am less concerned now with my child’s eating habits as I was in the past.	2.58 ± 1.239	3.26 ± 0.902	0.008
(22) I feel intervention for my child’s eating habits is important and needed soon.	2.51 ± 1.222	3.16 ± 1.252	0.016
(23) I feel intervention for my child’s eating is important but not needed at this time.	2.53 ± 1.077	2.84 ± 0.998	0.157
(24) I feel therapy intervention for my child’s eating habits is not needed.	3.14 ± 1.355	2.95 ± 1.344	0.517

## Data Availability

Not applicable.
